# Severe Burn Injury Alters Expression of Adrenergic Receptor Transcripts in a Rodent Model

**DOI:** 10.3390/ebj7030045

**Published:** 2026-08-21

**Authors:** Kristine Knappskog, Julia Kleinhapl, Titas Gladkauskas, Siren Fromreide, Dagrun Slettebø Daltveit, Jake E. Lowry, Per Morten Knappskog, Henning Onarheim, Anne Berit Guttormsen, Daniela Elena Costea, Amina El Ayadi, Juquan Song, Steven E. Wolf, Stian Kreken Almeland

**Affiliations:** 1 Research Group for Burn Care and Reconstructive Surgery, Department of Clinical Medicine, Faculty of Medicine, University of Bergen, 5009 Bergen, Norwayanne.guttormsen@uib.no (A.B.G.); stian.almeland@uib.no (S.K.A.); 2 Department of Surgery, The University of Texas Medical Branch, Galveston, TX 77555, USA; jukleinh@utmb.edu (J.K.); amelayad@utmb.edu (A.E.A.); jusong@utmb.edu (J.S.); swolf@utmb.edu (S.E.W.); 3 Norwegian National Burn Center, Department of Plastic, Hand, and Reconstructive Surgery, Haukeland University Hospital, 5021 Bergen, Norway; 4 Department of Medical Genetics, Haukeland University Hospital, 5021 Bergen, Norway; titas.gladkauskas@uib.no (T.G.); per.knappskog@uib.no (P.M.K.); 5 Division of Plastic, Aesthetic and Reconstructive Surgery, Department of Surgery, Medical University of Graz, 8010 Graz, Austria; 6 Department of Clinical Medicine, Faculty of Medicine, University of Bergen, 5020 Bergen, Norway; siren.fromreide@uib.no; 7 Centre for Cancer Biomarkers (CCBIO), University of Bergen, 5020 Bergen, Norway; daniela.costea@uib.no; 8 Department of Global Public Health and Primary Care, University of Bergen, 5020 Bergen, Norway; dagrun.daltveit@uib.no; 9 Animal Resource Center, The University of Texas Medical Branch at Galveston, Galveston, TX 77555, USA; jolowry@utmb.edu; 10 Department of Comparative Medicine, The University of Texas MD Anderson Cancer Center, Galveston, TX 77555, USA; 11 Department of Clinical Science, University of Bergen, 5021 Bergen, Norway; 12 Department of Anesthesiology and Intensive Care, Haukeland University Hospital, 5021 Bergen, Norway; 13 Department of Pathology, Haukeland University Hospital, 5021 Bergen, Norway

**Keywords:** burns, shock, resuscitation, norepinephrine, vasoconstrictor agents

## Abstract

Background: Norepinephrine is commonly used during acute burn resuscitation to maintain adequate perfusion pressure. However, its effects, together with the burn-induced catecholamine surge, on arterial α_1_-adrenergic receptors (α_1_-AR) remain unclear. This study examined whether burn injury and continuous norepinephrine infusion alter arterial α_1_-AR subtype expression at the transcriptional and protein levels. Methods: Sprague–Dawley rats were randomized to full-thickness scald burn involving 25% total body surface area with continuous intravenous norepinephrine or sodium chloride (NaCl) infusion, sham injury with norepinephrine infusion, or untreated controls. Mean arterial pressure (MAP) was measured at baseline and before euthanasia (six or 24 h). Aorta, carotid, and renal arteries were collected for analysis of α_1_-AR subtypes (*Adra1a*, *Adra1b*, and *Adra1d*) using quantitative PCR (qPCR) and immunohistochemistry (IHC). Results: Norepinephrine efficiently increased MAP in sham animals. In burned animals with norepinephrine, MAP increased at six hours (+10.6 mmHg) but fell below baseline at 24 h (−6.4 mmHg). Burned animals given NaCl had persistently lower MAP than those given norepinephrine, at six hours (−19.1 mmHg) and 24 h (−21.1 mmHg). qPCR demonstrated significant downregulation of *Adra1b* in the renal artery across all groups compared to control (7.8–16.4-fold, *p* < 0.001). Overall, changes in protein expression across different vascular beds and receptor subtypes were inconsistent. Conclusions: Norepinephrine increased MAP in both sham and burned animals, confirming the drug’s efficacy. Burn injury and sustained norepinephrine exposure were associated with early, vessel-specific alterations in α_1_-AR mRNA expression. The absence of consistent protein-level changes within 24 h suggests a delay in receptor expression on protein level.

## 1. Introduction

Severe burns induce a profound systemic inflammatory response accompanied by a massive increase in endogenous catecholamines, both at the burn site and systemically [1,2,3,4,5]. This catecholamine surge occurs within hours of injury as an integral part of the innate inflammatory response, and persists for months to years [2,3]. Due to potent cardiovascular effects, exogenous catecholamines, particularly epinephrine and norepinephrine, are commonly used as vasopressors to support blood pressure during acute treatment of burn injuries. In burn resuscitation, vasopressors are frequently administered during fluid therapy to maintain perfusion pressure and minimize risks of over-resuscitation. Although we find no clear international consensus on which vasopressors are preferred in burn shock, norepinephrine is the most commonly used [6,7].

Norepinephrine is a non-selective adrenergic receptor agonist and mediates vascular smooth muscle contraction primarily through α_1_-adrenergic receptor (α_1_-AR) activation [8,9,10]. As the primary mediators of catecholamine-induced increases in vascular resistance and arterial pressure, α_1_-ARs play a central role in blood pressure regulation. The α_1_-ARs have three known subtypes, α_1A_, α_1B_, and α_1D_, which differ in distribution across vascular beds [11,12]. Preclinical studies have suggested reduced vascular responsiveness to adrenergic receptor agonists after severe burn at six and 24 h after injury [13,14]. These observations raise the question of whether the endogenous catecholamine surge following severe burn injury interferes with adrenergic receptor sensitivity and potentially receptor expression.

While the hemodynamic effects of norepinephrine as a vasopressor are well established, its impact on adrenergic receptor regulation during the acute catecholamine surge following severe burn injury remains poorly understood [15,16]. In particular, there is limited direct evidence addressing whether exogenous norepinephrine administration during early burn resuscitation modifies α_1_-AR expression in systemic arteries. This study aims to characterize α_1_-AR expression in vascular tissues during the early resuscitation phase following severe burn injury and treatment with norepinephrine infusion in a rat model.

## 2. Materials and Methods

### 2.1. Animals

Thirty-six male Sprague–Dawley rats (350 g body weight; Charles River Laboratories, Wilmington, MA, USA) were randomly assigned to burn, sham, or control groups. Animals were housed in a 12 h light/dark cycle and acclimated for one week prior to the experiment. Standard rodent chow and water were provided ad libitum. The animals were housed individually from the onset of the study. The study was approved by the Institutional Animal Care and Use Committee of the University of Texas Medical Branch, Galveston (APN#2310056), and was performed in accordance with the Animal Research Reporting In vivo Experiments (ARRIVE) guidelines [17]. The animal experiments were initiated in January 2024. An overview of the experimental groups is available in Table 1.

### 2.2. Femoral Artery and Vein Catheterization

Femoral artery and vein catheterization was performed ipsilaterally for both burn and sham groups, following a procedure described by Jespersen et al., under deep isoflurane anesthesia (1.5–3% in 100% oxygen) [18]. Tapered polyurethane femoral vein catheters (SAI Infusion Technologies, Lake Villa, IL, USA) and femoral arterial catheters (Access Technologies, Skokie, IL, USA) were inserted into the femoral vein and artery and advanced into the abdominal vena cava and abdominal aorta, respectively, under sterile conditions. The catheters were filled with heparin in glycerol (500 IU/mL heparin in 50% glycerol) to ensure patency. Buprenorphine HCl 0.05 mg/kg was administered subcutaneously between the scapulae before surgery and repeated every 6–10 h until the burn procedure. Additional doses were administered if indicated based on clinical pain assessment. Postoperatively, animals were monitored at least twice daily. A 48 h recovery period was provided before the burn procedure.

### 2.3. Burn Procedure and Resuscitation

Under deep isoflurane anesthesia (3% in 100% oxygen), animals were secured in a template burn device to expose the shaved back and abdomen, which were then immersed in 96–100 °C water for 10 and 2 s, respectively, creating a full-thickness scald burn of approximately 25% TBSA. The skin was immediately dried with a clean tissue, and the animals were put on a heating pad to prevent hypothermia. The burned surface area was calculated using the Meeh formula with a rat-specific constant (k = 9.83), as described by Gouma et al., to adjust fluid resuscitation volumes [19]. Buprenorphine HCl 0.05 mg/kg was administered subcutaneously between the scapulae 30 min before the burn and repeated every 6–10 h thereafter until euthanasia. The sham-burn group underwent the same procedure, including administration of buprenorphine, except for boiling water immersion.

All burned animals were fluid resuscitated with lactated Ringer’s solution as suggested by the Parkland formula (4 mL/kg/%TBSA). Approximately 30 min after the burn, half the volume was administered intraperitoneally (IP), and the remaining volume was administered at eight hours after the burn. Forty-five minutes after burn, an infusion of either norepinephrine (16 µg/mL) diluted in 0.9% sodium chloride (NaCl) with 5% dextrose, or 0.9% NaCl alone, was initiated in awake animals using Genie Plus syringe pumps (Kent Scientific, Torrington, CT, USA). The norepinephrine infusion was titrated from 0.1 µg/kg/min to 0.3 µg/kg/min over one hour, with a similar increase in infusion rate for the NaCl control group. The norepinephrine dose was selected based on pilot experiments demonstrating that this infusion rate produced a sustained increase in mean arterial pressure in both burned and unburned rats and was well tolerated during prolonged infusion in awake animals. To maintain the planned resuscitation volume, the NaCl infusion volume was subtracted from the total IP lactated Ringer’s fluid volume. The infusions continued until the animals were euthanized, induced by isoflurane overdose, followed by exsanguination, at six or 24 h after burn. The norepinephrine solution was changed at 12 h to ensure the potency of the drug.

### 2.4. Blood Sampling and Invasive Arterial Pressure Monitoring

Blood samples were obtained from the femoral vein catheter into EDTA- and heparin-coated tubes at baseline (before burn or sham procedure) and prior to euthanasia. Hematological analyses, including white blood cell count with differential, hemoglobin, and hematocrit, were performed on EDTA-anticoagulated whole blood using an automated hematology analyzer (VETSCAN HM5, Zoetis, Parsippany, NJ, USA). Biochemical analyses, including albumin, total protein, and creatinine, were performed using an automated biochemistry analyzer (VetScan VS2, Zoetis). These parameters were selected to assess the systemic inflammatory response, hydration status, and renal function. Invasive arterial pressure was measured through the femoral artery catheter under light anesthesia (1–2% isoflurane in 100% oxygen) at baseline, before the second dose of LR, and before euthanasia, with ongoing norepinephrine or NaCl infusions. Arterial pressure was measured using the PoweLab/4SP (AD Instruments, Sydney, Australia), MLT0699 Disposable Blood Pressure Transducer (AD Instruments), and the LabChart software (version 7.3, AD Instruments).

### 2.5. Tissue Harvesting and Preparation

The aorta, both common carotids, and both renal arteries were harvested immediately after euthanasia. Tissues were rinsed with phosphate-buffered saline (PBS) to remove residual blood. The arteries designated for RNA extraction were immediately snap-frozen in liquid nitrogen and stored at −80 °C. Arteries intended for immunohistochemistry were fixed in 10% neutral-buffered formalin, then transferred to 70% ethanol, processed through graded alcohols, then xylene, and embedded in paraffin.

### 2.6. RNA Extraction and Quantitative Real-Time PCR

The arteries were disrupted by mechanical shearing using stainless steel beads (5 mm) and the TissueLyser II (Qiagen, Hilden, Germany). Total RNA was purified using the RNeasy Fibrous Tissue Mini Kit (Qiagen), according to the manufacturer’s protocol. Genomic DNA was removed by on-column DNase I digestion. RNA concentration and purity were measured using a NanoDrop spectrophotometer 1000 (Thermo Fisher Scientific, Waltham, MA, USA), and RNA integrity was evaluated using the TapeStation System 4200 (Agilent Technologies, Santa Clara, CA, USA). cDNA synthesis was performed from 0.5–1.5 μg of total RNA using SuperScript™ VILO™ Master Mix (Thermo Fisher Scientific), following the manufacturer’s instructions. Gene expression was quantified by TaqMan-based qPCR using probes specific for *Adra1a*, *Adra1b*, *Adra1d*, and *18S* (FAM-labelled), and the reference gene *ACTB* (VIC-labelled) (all from Thermo Fisher Scientific, #Rn00567876_m1, #Rn01471343_m1, #Rn00577931_m1, #Rn00667869_m1, and #Hs99999901_s1). Pilot experiments with eukaryotic 18S rRNA confirmed that ACTB expression remained stable across tissue types and treatment groups, supporting its use as a reference gene. qPCR was performed on an ABI 7900HT Fast Real-Time PCR System (Applied Biosystems, Foster City, CA, USA), with all reactions run in technical duplicates. PCR reactions (10 μL) were prepared with 5 μL of TaqMan Universal Master Mix (Thermo Fisher Scientific), 0.5 μL of each probe, and 1 μL of cDNA. Thermal cycling was conducted with an initial activation at 50 °C for 2 min and denaturation at 95 °C for 10 min, followed by 40 cycles of 95 °C for 15 s and 60 °C for 1 min. Relative gene expression was calculated using the 2^−ΔΔCT^ method, normalized to ACTB [20]. All assays included no-template controls.

### 2.7. Immunohistochemistry

Immunohistochemistry was performed using Dako EnVision FLEX kit (Agilent Technologies, Santa Clara, CA, USA). Four µm-thick tissue sections were cut on a microtome and mounted on Superfrost Plus slides (Thermo Fisher Scientific). The tissue sections were baked at 60 °C for 1 h, deparaffinized with xylene, and rehydrated with decreasing concentrations of alcohol. Antigen retrieval was performed using Dako Target Retrieval solution pH 9 (Agilent Technologies) in a microwave for 25 min. Endogenous peroxidase activity was blocked with EnVision FLEX peroxidase blocking solution (Agilent Technologies) for 5 min. Unspecific binding sites were blocked using 10% Normal Goat Serum (Agilent Technologies) in 3% Bovine Serum Albumin (BSA, Sigma-Aldrich, St. Louis, MO, USA) for 60 min at room temperature (RT). Tissue sections were then incubated with primary antibody (Table 2) for 60 min at RT or at 4 °C overnight. This was followed by incubation with EnVision FLEX HRP secondary antibody (Agilent Technologies) for 30 min at RT, after which the antibody staining was visualized using 3,3′-diaminobenzidine (DAB) chromogen (Agilent Technologies) for 10 min. Tris-buffered saline with Tween (TBST) was used as a washing buffer between each step for 2 × 5 min. Finally, the sections were counterstained with 50% Gill’s Hematoxylin (Sigma-Aldrich) for 2 min, then dehydrated in alcohol and xylene and mounted under coverslips using Pertex (Histolab Products AB, Gothenburg, Sweden). Slides were scanned using a whole-slide digital scanner (Hamamatsu NanoZoomer XR, Shizuoka, Japan).

Immunohistochemical staining was quantified using QuPath (version 0.5.1) [21]. Whole-slide images were imported, and regions of interest were manually annotated to include the arterial wall while excluding the tunica adventitia and surrounding tissue. DAB-positive staining was identified using QuPath’s built-in color deconvolution and threshold-based detection. Manual optical density (OD) thresholds were defined on representative sections and adjusted while visually inspecting the entire arterial ring(s), and the exact OD value used was recorded. Quantification was performed blinded to group allocation to reduce bias. Detection parameters included a minimum object area of 20 µm^2^ and a hole-filling area up to 100 µm^2^. These settings were selected to minimize inclusion of background noise and small artifacts while preserving continuous DAB-positive staining within the arterial wall. The percentage of DAB-positive areas within each region of interest was calculated and used for statistical analysis. When available, two arterial sections per artery were analyzed, and the mean percentage of DAB-positive area across the two sections was used for statistical analysis.

### 2.8. Statistical Analysis

Mean arterial pressure (MAP) and lab values were analyzed using multilevel mixed-effect linear regression models with restricted maximum likelihood estimation. Fixed effects included time, group, and their interaction, with rat ID modeled as a random intercept. *p*-values and confidence intervals were adjusted for multiple comparisons using the Bonferroni method. qPCR and IHC data were collected once per animal at euthanasia and were analyzed using one-way analysis of variance (ANOVA) to compare the six experimental groups, with Bonferroni correction for multiple comparisons. Model assumptions were assessed by visual inspection of residual Q–Q plots. Results from the mixed-effects models were presented as means with 95% confidence intervals. qPCR and IHC results were presented as mean ± standard error of the mean (SEM). The observational time points at six and 24 h post-burn were selected to capture temporal changes during the acute resuscitation phase following severe burn injury.

## 3. Results

Mean body weight before sham or burn injury and mean percentage of total body surface area (%TBSA) burned did not differ among the groups (Table 3).

### 3.1. Mean Arterial Pressure and Laboratory Parameters

**Figure 1:**
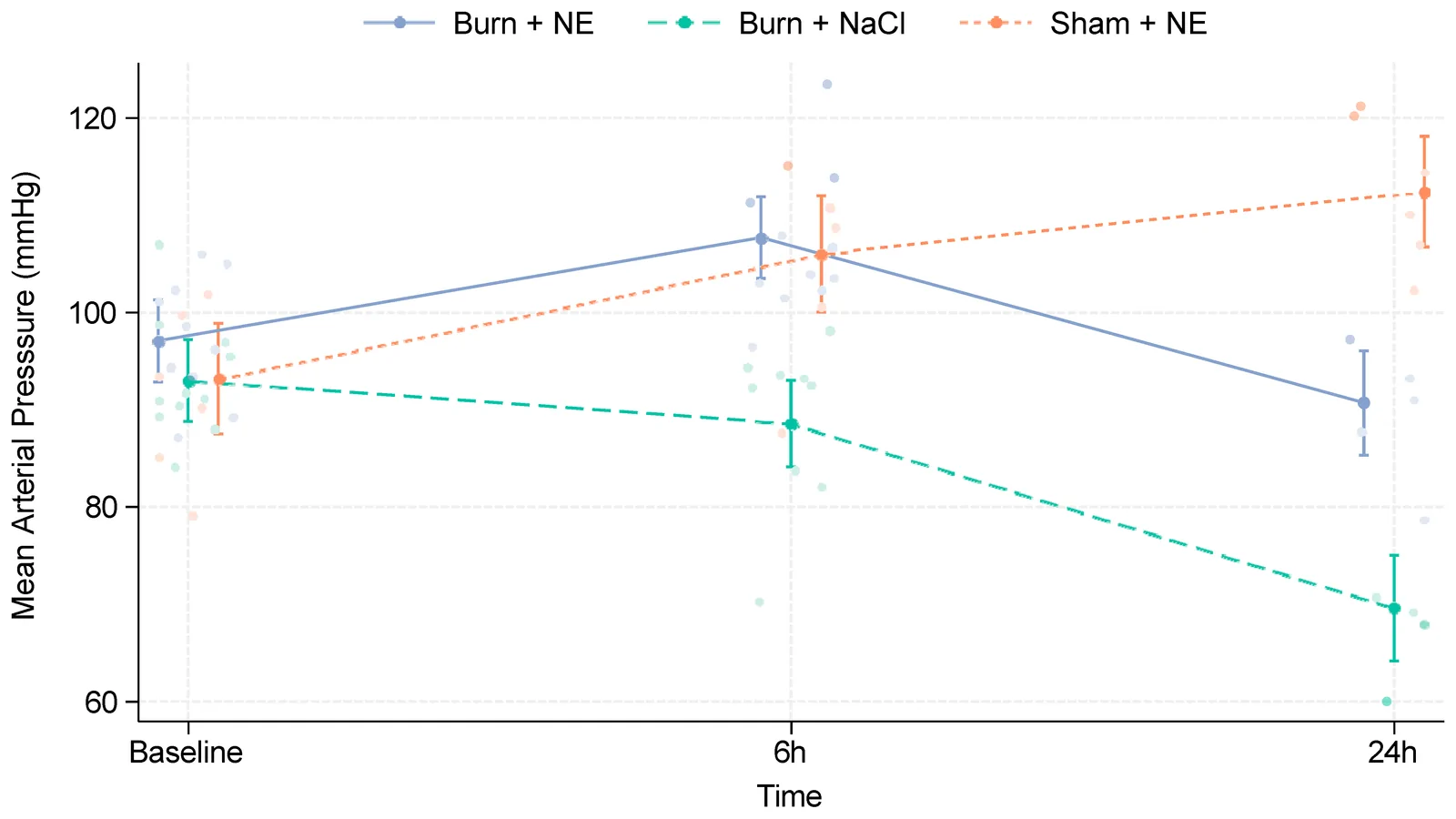
Mean arterial pressure values at baseline (before burn injury), six and 24 h after burn injury, in burned animals with Norepinephrine infusion (Burn + NE), burned animals with saline infusion (Burn + NaCl), and unburned animals with Norepinephrine infusion (Sham + NE). NE, norepinephrine; NaCl, sodium chloride; MAP, mean arterial pressure.

**Figure 2:**
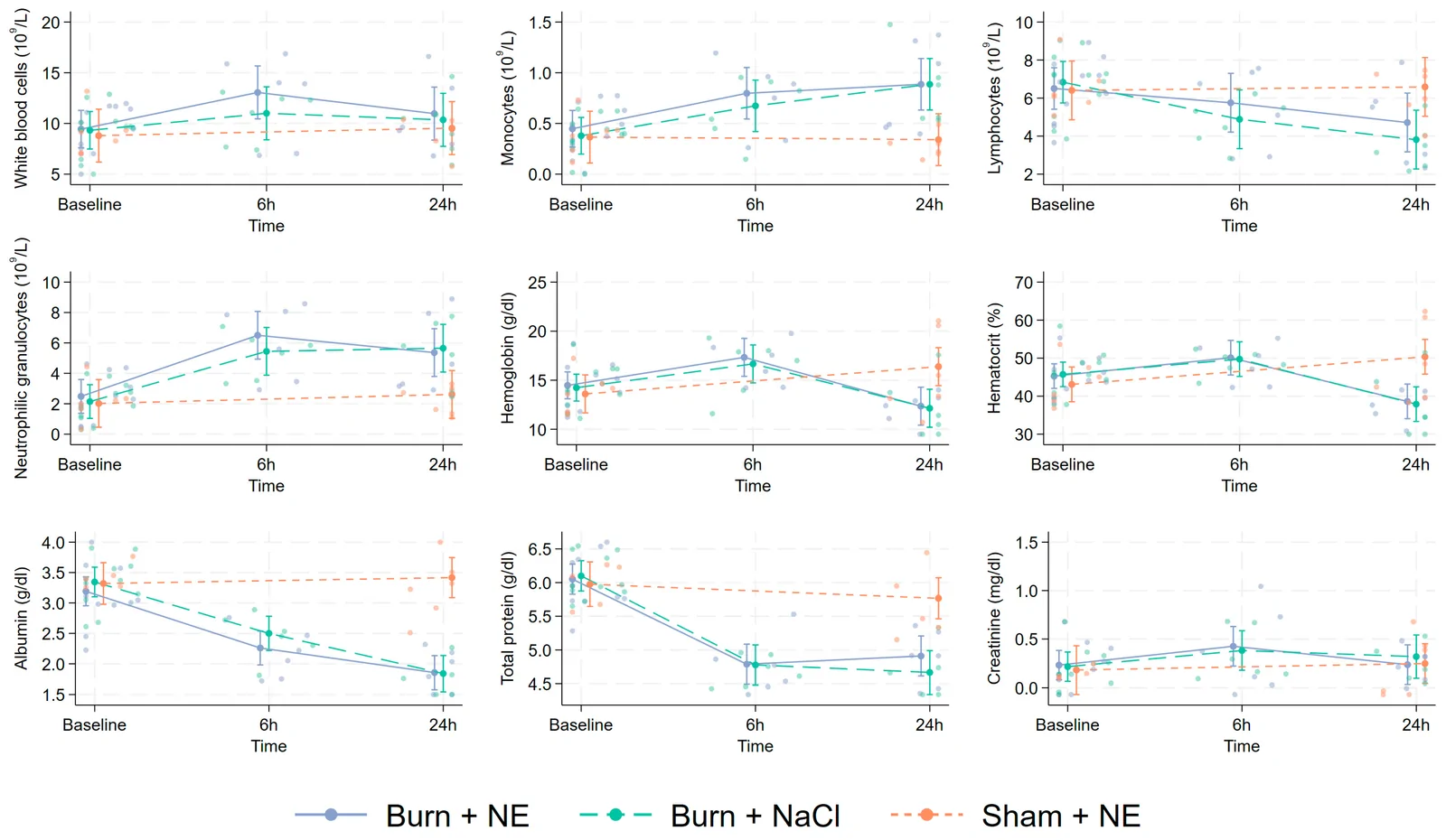
Hematological and biochemical parameters measured from blood samples obtained from femoral vein catheters at baseline (before burn or sham procedure) and prior to euthanasia. NE, norepinephrine; NaCl, sodium chloride.

MAP measurements are illustrated in Figure 1. At baseline, MAP was similar in all three groups with an average of 97.1 mmHg (95% CI: 92.9 to 101.3). MAP increased from baseline in the sham + norepinephrine group at both six and 24 h (12.8 mmHg, 95% CI: 6.4 to 19.3; 19.2 mmHg, 95%CI: 13.2 to 25.3). The burn + norepinephrine group MAP increased at six hours by 10.6 mmHg (95% CI: 6.1 to 15.1) relative to baseline but declined below baseline at 24 h (−6.4 mmHg; 95% CI: −12.4 to −0.3). In contrast, the burn + NaCl group exhibited a sustained decrease in MAP and remained significantly lower than the burn + norepinephrine group at both time points, with between-group differences of −19.1 mmHg (95% CI −26.6 to −11.7) at six hours and −21.1 mmHg (95% CI −30.4 to −11.8) at 24 h. Selected hematological and biochemical parameters are shown in Figure 2. Both neutrophilic granulocytes and monocytes increased after burn injury at both six and 24 h compared with baseline, while a reduction in serum albumin was observed at the same time points. No significant changes in creatinine levels were observed following burn injury.

### 3.2. Quantitative Real-Time PCR

**Figure 3:**
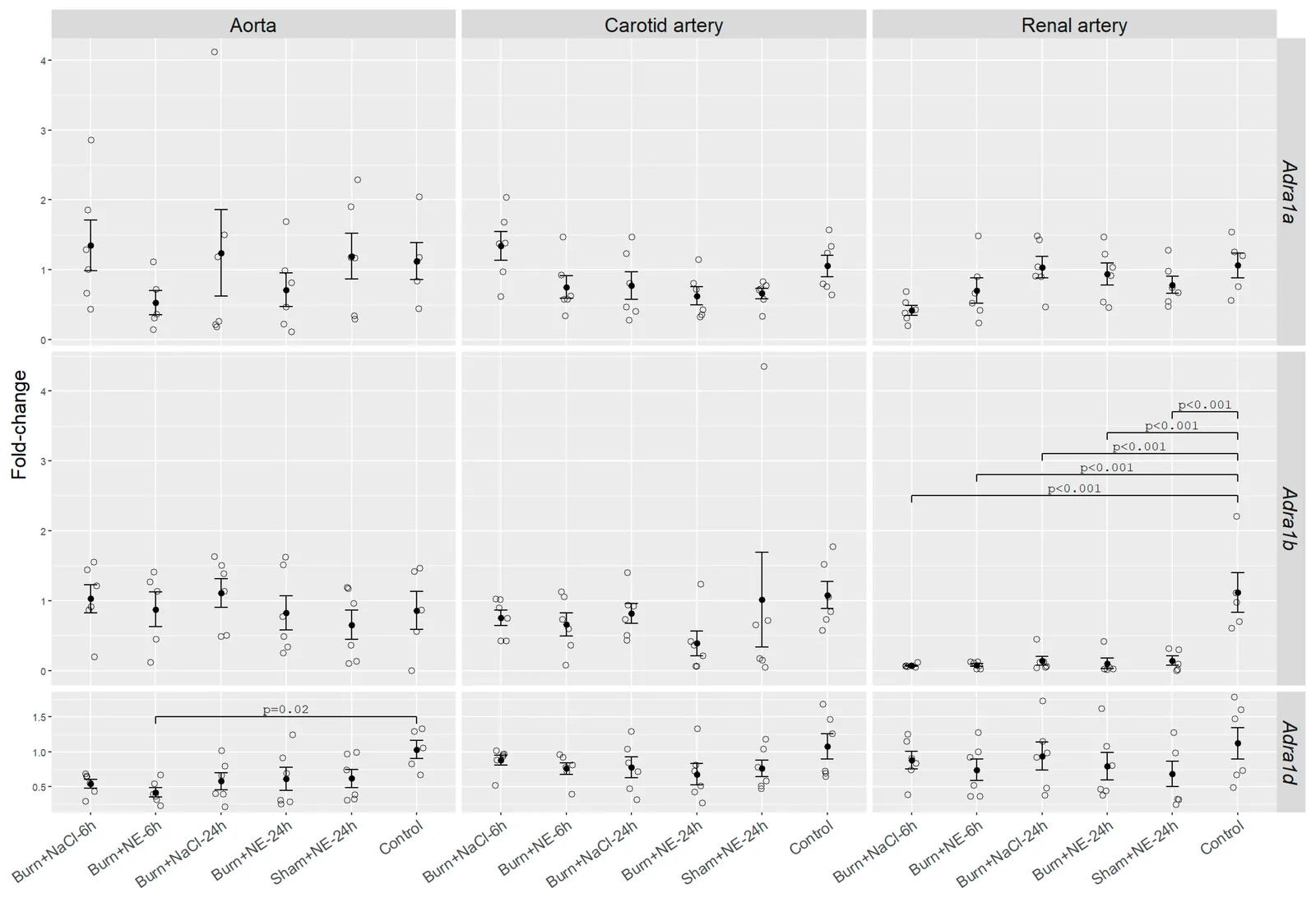
Relative expression of α_1_-adrenergic receptor subtypes *Adra1a*, *Adra1b*, and *Adra1d* in the aorta, carotid, and renal arteries. Gene expression was quantified by qPCR and normalized to the reference gene *ACTB*. Data are presented as fold change relative to the control group using the 2^−ΔΔCT^ method. Data were analyzed using one-way ANOVA followed by Bonferroni’s multiple comparisons test. NE, norepinephrine; NaCl, sodium chloride.

*Adra1a*, *Adra1b*, and *Adra1d* expression levels were quantified in the aorta, carotid, and renal arteries using quantitative polymerase chain reaction (qPCR). Results are presented as fold changes relative to the control group (Figure 3).

***Adra1a*.** The ANOVA indicated overall group differences in the carotid and renal arteries (*p* = 0.03 and *p* = 0.04, respectively), but Bonferroni-adjusted post hoc comparisons did not identify any significant pairwise differences in expression compared to control across all three arteries. A 2.1-fold decrease in the aorta was observed in the burn + norepinephrine group at six hours, and a 1.6-fold decrease at 24 h, though these changes were not statistically significant.

***Adra1b*.** A pronounced decrease compared to control was observed in the renal artery in all groups (7.8–16.4-fold, *p* < 0.001). In the carotid artery, a 2.8-fold reduction was observed in the burn + norepinephrine 24 h group, although this finding did not reach significance.

***Adra1d*.** In the aorta, *Adra1d* expression was significantly reduced in the burn + norepinephrine 6 h group compared with control (2.5-fold reduction, *p* = 0.02). The other treatment groups were downregulated 1.7–1.9-fold, but this was not significant. No differences between groups were found for the carotid and renal arteries.

### 3.3. Immunohistochemistry

**Figure 4:**
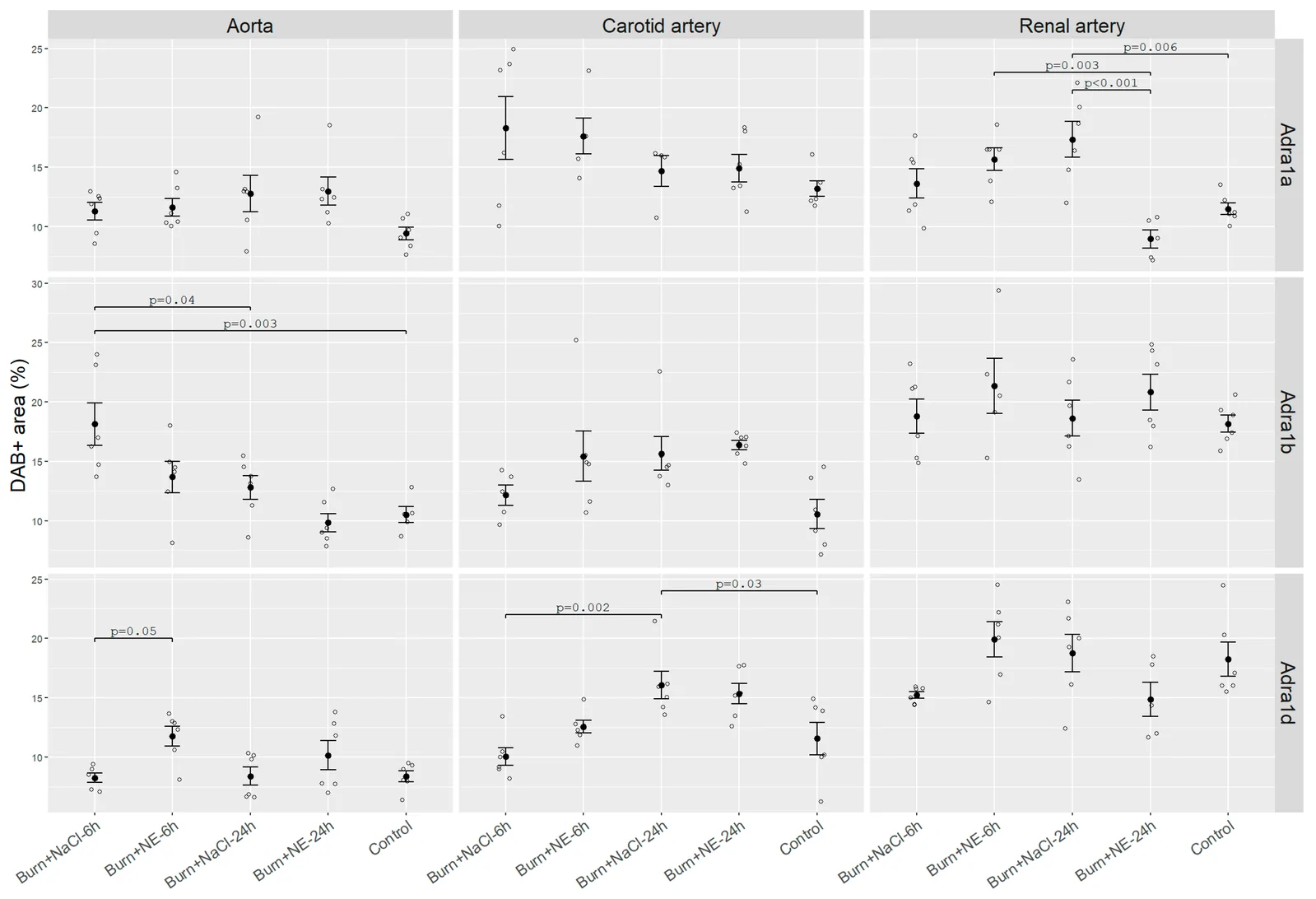
Immunohistochemical staining and quantification of percent DAB-positive area for α_1_-adrenergic receptor subtypes *Adra1a*, *Adra1b*, and *Adra1d* in the aorta, carotid artery, and renal artery. DAB-positive area is expressed as a percentage of the total arterial wall area, excluding the tunica adventitia. Data are presented as mean ± SEM. Statistical analysis was performed using one-way ANOVA followed by Bonferroni’s multiple-comparisons test. Significance was set at *p* < 0.05. NE, norepinephrine; NaCl, sodium chloride.

Immunohistochemistry (IHC) was used to quantify protein expression of the α_1_-AR subtypes (*Adra1a*, *Adra1b*, and *Adra1d*) in selected arteries at six and 24 h after burn injury with norepinephrine or NaCl administration, compared with non-burned controls. Results are reported as the percentage of DAB-positive area of the arterial wall excluding the tunica adventitia (Figure 4).

***Adra1a*:** In the renal artery, the burn + norepinephrine-24 h group showed reduced *Adra1a* DAB-positive area and was significantly lower than both the burn + NaCl 24 h group (9.0% vs. 17.3%, *p* = 0.003) and the burn + norepinephrine 6 h group (9.0% vs. 15.7%, *p* = 0.003). In contrast, the burn + NaCl 24 h group demonstrated increased *Adra1a* staining compared with controls (17.3% vs. 11.5%, *p =* 0.006). No significant differences were observed in the other arteries.

***Adra1b*:** In the aorta, *Adra1b* DAB-positive staining was lower in the burn + NaCl group at 24 h than at 6 h (12.8 vs. 18.1%, *p =* 0.04). A similar pattern was observed for the burn + norepinephrine group, but this finding did not reach significance (9.8 vs. 13.7, *p* = 0.32). However, besides the burn + NaCl 6 h group, which was above control (*p =* 0.003), none of the other groups were significantly different from control. In the carotid artery, the staining varied by group (*p* = 0.02), but after adjustment for multiple comparisons, none of the pairwise comparisons were statistically different. No significant differences were observed in the renal artery.

***Adra1d*:** In the carotid artery, the burn + NaCl 24 h group showed increased *Adra1d* DAB-positive area compared with controls (16.1% vs. 11.5%, *p* = 0.03) and the Burn+ NaCl 6 h group (16.1% vs. 10.0%, *p* = 0.002). No significant differences were observed in the other groups in the carotid artery. In the renal artery, we found statistical differences among the groups (*p* = 0.05), but none of the pairwise comparisons were statistically different.

## 4. Discussion

This study characterizes α_1_-adrenergic receptor expression during the early resuscitation phase following severe burn injury. Severe burn injury was induced using a validated animal model and confirmed by %TBSA burned and laboratory findings of a systemic inflammatory response, including altered inflammatory markers and reduced serum albumin (Figure 2) [22]. Hemodynamic data further demonstrated that the administered norepinephrine dose effectively increased mean arterial pressure in both burned and unburned animals (Figure 1), confirming the intended treatment effect of norepinephrine in burn resuscitation. At the transcript level, qPCR revealed a pronounced change in the renal artery, where *Adra1b* expression decreased 7.8–16.4-fold compared to control (Figure 3). At the protein level, the most pronounced changes were limited to *Adra1a* in the renal artery, where burn + norepinephrine 24 h was below burn + NaCl 24 h and burn + norepinephrine 6 h (Figure 4). However, IHC analysis did not reveal a consistent staining pattern across α_1_-AR subtypes.

Hypotension after burn injury is multifactorial and reflects plasma loss caused by increased microvascular permeability, combined with myocardial depression and reduced cardiac output [23]. Impaired vascular responsiveness, possibly due to alterations in vascular smooth muscle receptor activity, has been less discussed as a contributing factor [14,24]. α_1_-ARs can be regulated through several mechanisms, including receptor desensitization and changes in RNA and protein expression [25,26]. Although changes in responsiveness to norepinephrine have been observed after burn, the mechanisms underlying these changes remain unknown [13]. Desensitization, through receptor phosphorylation or internalization, typically occurs after sustained agonist exposure [25,27,28,29]. The burn-induced catecholamine surge may therefore partly account for the reduced vascular responsiveness to adrenergic receptor agonists seen after burn injury [13,14,30]. In a preclinical model, Evans found a reduced potency of the selective α_1_-AR agonist, phenylephrine, to increase MAP 24 h after burn injury [14]. However, reactivity to phenylephrine in isolated resistance arteries at the same time point was unchanged, supporting the theory that the post-burn catecholamine surge contributes to receptor desensitization.

In our study, in vivo vascular responsiveness could not be directly assessed, as only a continuous norepinephrine infusion was used, and comparisons were only between groups and not within animals. In addition, MAP measurements were obtained under isoflurane anesthesia, which may have influenced the absolute MAP values. Although all measurements were performed under standardized isoflurane anesthesia using the same anesthetic protocol and concentrations across all groups, a cumulative effect of prolonged anesthesia at the later time points cannot be excluded and may have influenced the absolute MAP values. Within these limitations, when comparing burned animals, norepinephrine had a similar effect on MAP compared to the NaCl groups at six and 24 h after injury. In contrast, when compared with sham animals, burned animals receiving norepinephrine maintained similar MAP values at six hours. At 24 h, MAP in burned animals declined below baseline, while MAP in the sham group increased further with norepinephrine infusion. Hemoglobin and hematocrit values at 24 h were consistent with adequate fluid resuscitation, suggesting that factors other than hypovolemia contributed to the hypotension. However, the present study was not designed to address these mechanisms directly, and the findings require further validation, including ex vivo vascular reactivity studies with concentration–response curves to adrenergic receptor agonists.

A transient decrease in α_1_-AR mRNA has been reported within hours of norepinephrine stimulation [31]. In line with this, our data demonstrated an overall trend toward reduced α_1_-AR transcript levels after burn injury across the carotid, aorta, and renal artery, although not all groups reached statistical significance. Burn injured animals receiving norepinephrine often showed a tendency toward lower expression compared with NaCl-treated burn groups, although these differences were modest and inconsistent. Notably, transcriptional trends of downregulation were also observed in sham + norepinephrine animals, suggesting a specific effect of norepinephrine on the receptor expression level independent of thermal injury. This indicates that the administered norepinephrine dose may have been sufficient to alter α_1_-AR expression alone, and that, in the case of burn injury, norepinephrine treatment may augment the downregulation of α_1_-AR already caused by the endogenous catecholamine release. However, these observations should be interpreted in light of the study design. The primary objective of this study was to investigate the effect of norepinephrine during early burn resuscitation. However, the absence of a Sham + NaCl 24 h group limits our ability to fully distinguish the independent effects of burn injury, surgery, and norepinephrine infusion on α_1_-AR expression.

Protein staining of α_1_-AR assessed by IHC showed inconsistent variation across groups for the different α_1_-AR subtypes. Given the observed downregulations at the transcript level, corresponding reductions at the protein level might have been expected. In the renal artery, reduced staining was observed for *Adra1a* in burned animals receiving norepinephrine at 24 h compared with burned animals that received a control infusion with NaCl. Interestingly, no differences were seen on protein level for *Adra1b* in the renal artery. Protein studies have found the α_1_-ARs half-life to be between 7–39 h, depending on the subtype [32,33,34,35,36]. This may partly explain why group differences in protein staining did not correlate with the mRNA expression in our study, as the final time point was 24 h. While qPCR reflects changes in gene transcription, IHC captures receptor protein abundance and localization, which may be influenced by translation, intracellular trafficking, and degradation, particularly under sustained catecholamine exposure. Additionally, α_1_-ARs are expressed not only in vascular smooth muscle cells, but also in endothelial cells and adventitial components. In the present study, immunoreactivity was assessed across the vessel wall, excluding the tunica adventitia, and as a result, layer-specific changes in α_1_-AR expression may have been missed, potentially contributing to the heterogeneous staining patterns observed across receptor subtypes and vascular beds. Consequently, the present protein-level findings should be interpreted as reflecting integrated changes across vascular cell types rather than isolated endothelial or smooth muscle effects. Layer-specific analyses could further distinguish changes in vascular smooth muscle and endothelial cells. Moreover, immunohistochemical quantification remains semi-quantitative and subject to methodological variability, which should be considered when interpreting differences in positive staining area. The use of more quantitative and receptor-specific protein detection methods, assessment of time points beyond 24 h, and application of layer-specific or multiplexed approaches to resolve cell-type–specific α_1_-AR regulation may help identify changes in α_1_-AR protein expression after burn injury that were not detectable with the present study design. Increased knowledge of receptor expression changes after burns may help guide vasopressor therapy in burn resuscitation.

Although we observed a general trend toward mRNA downregulation, variations were observed across the three arteries and α_1_-AR subtypes. Both α_1A_- and α_1D_-AR are mediators of smooth muscle contraction, and important for blood pressure control [9,10]. α_1B_-AR has been described as mainly regulatory or trophic, with potential roles in surface expression of other α_1_-AR and in vascular remodeling, and may also contribute to blood pressure regulation [9,37,38,39]. The most prominent finding in our study was the downregulation of *Adra1b* mRNA in the renal artery. Although the receptor’s function in this artery is not fully defined, altered signaling could be relevant for renal perfusion and blood pressure regulation. Acute kidney injury has been identified as a potential risk associated with vasopressor use during the early burn resuscitation phase [40]. However, the interplay among the different α_1_-ARs remains poorly understood, complicating the interpretation of transcript changes and their specific impact on vascular tone.

Relying on vasopressor monotherapy in shock management may be problematic [16]. Catecholamine-resistant shock remains a challenge in intensive care patients, and increasing doses of adrenergic receptor agonists have been associated with increased mortality in this patient population [41,42,43,44]. Catecholamine resistance in burn shock has not been thoroughly investigated, although recent findings from experimental models suggest reduced responsiveness to adrenergic receptor agonists in the early post-burn period [13,14]. In contrast, vasopressin responsiveness seems to be preserved or even enhanced after burn [14]. Similar findings have been described in septic shock, with reduced vascular responsiveness to norepinephrine, while intact to vasopressin [45,46]. Comparable trends have also been reported in burn patients with septic shock, suggesting possible parallels in receptor regulation during systemic inflammatory stress across different etiologies [30]. Although norepinephrine had the intended effects on blood pressure in our study, there are seemingly trade-offs associated with monotherapeutic targeting. Thus, combination therapy with vasopressors targeting different receptor systems might prove beneficial and should be the focus of future research.

## 5. Conclusions

This is the first study to investigate adrenergic receptor transcript expression in vascular smooth muscle during the early burn resuscitation phase. We observed an early downregulation of α_1_-adrenergic receptor transcripts as early as six hours after burn injury, whereas protein expression did not show a consistent pattern across α_1_-AR subtypes during the 24 h observation period, potentially reflecting differences between transcriptional regulation and protein turnover. Although these findings do not demonstrate altered receptor function or vascular responsiveness, they provide novel insight into the early molecular regulation of vascular adrenergic receptors following severe burn injury. Furthermore, they establish a molecular basis for investigating whether altered α_1_-adrenergic receptor expression contributes to changes in vascular responsiveness during burn resuscitation.

## Figures and Tables

**Table 1:** Experimental groups and analyses performed.

Group	*n*	Burn Injury	Intravenous Infusion	Time of Euthanasia	Analysis Performed
Burn + NaCl—6 h	6	Yes	NaCl	6 h post-burn	MAP, qPCR, IHC
Burn + NE—6 h	6	Yes	NE	6 h post-burn	MAP, qPCR, IHC
Burn + NaCl—24 h	6	Yes	NaCl	24 h post-burn	MAP, qPCR, IHC
Burn + NE—24 h	6	Yes	NE	24 h post-burn	MAP, qPCR, IHC
Sham + NE—24 h	6	No	NE	24 h post-sham	MAP, qPCR
Control	6	No	None	Not applicable	qPCR, IHC

NE, norepinephrine; NaCl, sodium chloride; MAP, mean arterial pressure; qPCR, quantitative polymerase chain reaction; IHC, immunohistochemistry.

**Table 2:** Primary antibodies used for immunohistochemistry.

Target (Protein)	Class	Clone	Host	Supplier	Catalog No.	Working Dilution	Incubation
*Adra1a* (α1A-AR)	Recombinant monoclonal	JE62-50	Rabbit	Invitrogen	MA5-44824	1:300	60 min at RT
*Adra1b* (α1B-AR)	Polyclonal	N/A	Rabbit	Proteintech	19776-1-AP	1:500	60 min at RT
*Adra1d* (α1D-AR)	Polyclonal	N/A	Rabbit	Invitrogen	PA5-72171	1:200	Overnight at 4 °C

RT, room temperature.

**Table 3:** Baseline characteristics of the study groups. Mean body weight before burn injury and total body surface area (TBSA) burned, expressed as percentages, are presented. No significant differences were observed between the groups for body weight (*p* = 0.59) or percentage TBSA burned (*p* = 0.14).

Characteristics		
Group	Weight Before Burn (g), Mean (SD)	TBSA Burned (%), Mean (SD)
Burn + NE-6 h	350 (22.1)	26 (2.3)
Burn + NE-24 h	343 (15.5)	26 (1.6)
Burn + NaCl-24 h	351 (16.8)	24 (0.7)
Burn + NaCl-6 h	365 (15.8)	25 (0.9)
Control	357 (23.5)	-
Sham + NE-24 h	350 (31.2)	-

NE, norepinephrine; NaCl, sodium chloride; TBSA, total body surface area; SD, standard deviation.

## Data Availability

The datasets generated and/or analyzed during the current study are available from the corresponding author upon reasonable request.

## Abbreviations

The following abbreviations are used in this manuscript:

ACTB	Beta-actin
ANOVA	Analysis of variance
ARRIVE	Animal Research: Reporting of In Vivo Experiments
BSA	Bovine serum albumin
DAB	3,3′-Diaminobenzidine
IHC	Immunohistochemistry
IP	Intraperitoneal
MAP	Mean arterial pressure
NaCl	Sodium chloride
NE	Norepinephrine
OD	Optical density
PBS	Phosphate-buffered saline
qPCR	Quantitative polymerase chain reaction
RT	Room temperature
TBSA	Total body surface area
TBST	Tris-buffered saline with Tween
α_1_-AR	Alpha-1 adrenergic receptor

## References

[1] Goodall M., Stone C., Haynes B.W.J. (1957). Urinary output of adrenaline and noradrenaline in severe thermal burns. Ann. Surg..

[2] Kulp G.A., Herndon D.N., Lee J.O., Suman O.E., Jeschke M.G. (2010). Extent and magnitude of catecholamine surge in pediatric burned patients. Shock.

[3] Norbury W.B., Herndon D.N., Branski L.K., Chinkes D.L., Jeschke M.G. (2008). Urinary cortisol and catecholamine excretion after burn injury in children. J. Clin. Endocrinol. Metab..

[4] Sivamani R.K., Pullar C.E., Manabat-Hidalgo C.G., Rocke D.M., Carlsen R.C., Greenhalgh D.G., Isseroff R.R. (2009). Stress-mediated increases in systemic and local epinephrine impair skin wound healing: Potential new indication for beta blockers. PLoS Med..

[5] Matsui M., Kudo T., Kudo M., Ishihara H., Matsuki A. (1991). The endocrine response after burns. Agressologie.

[6] Soussi S., Berger M.M., Colpaert K., Dünser M.W., Guttormsen A.B., Juffermans N.P., Knape P., Koksal G., Lavrentieva A. (2018). Hemodynamic management of critically ill burn patients: An international survey. Crit. Care.

[7] Cartotto R., Johnson L.S., Savetamal A., Greenhalgh D., Kubasiak J.C., Pham T.N., A Rizzo J., Sen S., Main E. (2024). American Burn Association clinical practice guidelines on burn shock resuscitation. J. Burn Care Res..

[8] Aboud R., Shafii M., Docherty J.R. (1993). Investigation of the subtypes of alpha 1-adrenoceptor mediating contractions of rat aorta, vas deferens and spleen. Br. J. Pharmacol..

[9] Daly C.J., Deighan C., McGee A., Mennie D., Ali Z., McBride M., McGrath J.C. (2002). A knockout approach indicates a minor vasoconstrictor role for vascular α 1B-adrenoceptors in mouse. Physiol. Genom..

[10] Docherty J.R. (2011). Vasopressor nerve responses in the pithed rat, previously identified as α2-adrenoceptor mediated, may be α1D-adrenoceptor mediated. Eur. J. Pharmacol..

[11] Docherty J.R. (2010). Subtypes of functional α1-adrenoceptor. Cell. Mol. Life Sci..

[12] Martí D., Miquel R., Ziani K., Gisbert R., Ivorra M.D., Anselmi E., Moreno L., Villagrasa V., Barettino D., D’OCon P. (2005). Correlation between mRNA levels and functional role of alpha1-adrenoceptor subtypes in arteries: Evidence of alpha1L as a functional isoform of the alpha1A-adrenoceptor. Am. J. Physiol. Heart Circ. Physiol..

[13] DeSpain K., Rosenfeld C.R., Huebinger R., Wang X., Jay J.W., Radhakrishnan R.S., Wolf S.E., Song J. (2021). Carotid smooth muscle contractility changes after severe burn. Sci. Rep..

[14] Evans A.E., Vana P.G., LaPorte H.M., Kennedy R.H., Gamelli R.L., Majetschak M. (2017). Cardiovascular responsiveness to vasopressin and α1-adrenergic receptor agonists after burn injury. J. Burn Care Res..

[15] Hamzaoui O., Jozwiak M., Geffriaud T., Sztrymf B., Prat D., Jacobs F., Monnet X., Trouiller P., Richard C., Teboul J. (2018). Norepinephrine exerts an inotropic effect during the early phase of human septic shock. Br. J. Anaesth..

[16] Leone M., Einav S., Antonucci E., Depret F., Lakbar I., Martin-Loeches I., Wieruszewski P.M., Myatra S.N., Khanna A.K. (2023). Multimodal strategy to counteract vasodilation in septic shock. Anaesth. Crit. Care Pain Med..

[17] du Sert N.P., Hurst V., Ahluwalia A., Alam S., Avey M.T., Baker M., Browne W.J., Clark A., Cuthill I.C., Dirnagl U. (2020). The ARRIVE guidelines 2.0: Updated guidelines for reporting animal research. PLoS Biol..

[18] Jespersen B., Knupp L., Northcott C.A. (2012). Femoral arterial and venous catheterization for blood sampling, drug administration and conscious blood pressure and heart rate measurements. J. Vis. Exp..

[19] Gouma E., Simos Y., Verginadis I., Lykoudis E., Evangelou A., Karkabounas S. (2012). A simple procedure for estimation of total body surface area and determination of a new value of Meeh’s constant in rats. Lab. Anim..

[20] Livak K.J., Schmittgen T.D. (2001). Analysis of relative gene expression data using real-time quantitative PCR and the 2^−ΔΔCT^ method. Methods.

[21] Bankhead P., Loughrey M.B., Fernández J.A., Dombrowski Y., McArt D.G., Dunne P.D., McQuaid S., Gray R.T., Murray L.J., Coleman H.G. (2017). QuPath: Open source software for digital pathology image analysis. Sci. Rep..

[22] Barrow R.E., Meyer N.A., Jeschke M.G. (2001). Effect of varying burn sizes and ambient temperature on the hypermetabolic rate in thermally injured rats. J. Surg. Res..

[23] Causbie J.M., Sattler L.A., Basel A.P., Britton G.W., Cancio L.C. (2021). State of the art: An update on adult burn resuscitation. Eur. Burn J..

[24] Cioffi W.G., DeMeules J.E., Gamelli R.L. (1988). Vascular reactivity following thermal injury. Circ. Shock.

[25] Akinaga J., Lima V., Kiguti L.R.d.A., Hebeler-Barbosa F., Alcántara-Hernández R., García-Sáinz J.A., Pupo A.S. (2013). Differential phosphorylation, desensitization, and internalization of *α*1A−adrenoceptors activated by norepinephrine and oxymetazoline. Mol. Pharmacol..

[26] Michelotti G.A., Price D.T., Schwinn D.A. (2000). α1-Adrenergic receptor regulation: Basic science and clinical implications. Pharmacol. Ther..

[27] Alfonzo-Méndez M.A., Carmona-Rosas G., Hernández-Espinosa D.A., Romero-Ávila M.T., García-Sáinz J.A. (2018). Different phosphorylation patterns regulate α1D-adrenoceptor signaling and desensitization. Biochim. Biophys. Acta (BBA)-Mol. Cell Res..

[28] García-Sáinz J.A., Romero-Ávila M.T., Alcántara-Hernández R. (2011). Mechanisms involved in α1B-adrenoceptor desensitization. IUBMB Life.

[29] Price R.R., Morris D.P., Biswas G., Smith M.P., Schwinn D.A. (2002). Acute agonist-mediated desensitization of the human α1a-adrenergic receptor is primarily independent of carboxyl terminus regulation. J. Biol. Chem..

[30] Cartotto R., McGibney K., Smith T., Abadir A. (2007). Vasopressin for the septic burn patient. Burns.

[31] Izzo N.J., E Seidman C., Collins S., Colucci W.S. (1990). Alpha 1-adrenergic receptor mRNA level is regulated by norepinephrine in rabbit aortic smooth muscle cells. Proc. Natl. Acad. Sci. USA.

[32] Hughes R.J., Insel P.A. (1986). Agonist-mediated regulation of alpha 1- and beta 2-adrenergic receptor metabolism in a muscle cell line, BC3H-1. Mol. Pharmacol..

[33] Izzo N.J., Colucci W.S. (1994). Regulation of alpha 1B-adrenergic receptor half-life: Protein synthesis dependence and effect of norepinephrine. Am. J. Physiol..

[34] Mauger J.P., Sladeczek F., Bockaert J. (1982). Characteristics and metabolism of alpha 1 adrenergic receptors in a nonfusing muscle cell line. J. Biol. Chem..

[35] Sallés J., Gascón S., Badia A. (1996). Sustained increase in rat myocardial alpha 1A-adrenoceptors induced by 6-hydroxydopamine treatment involves a decelerated receptor turnover. Naunyn. Schmiedeberg’s Arch. Pharmacol..

[36] Sladeczek F., Bockaert J. (1983). Turnover in vivo of alpha 1-adrenergic receptors in rat submaxillary glands. Mol. Pharmacol..

[37] Hague C., Uberti M.A., Chen Z., Hall R.A., Minneman K.P. (2004). Cell surface expression of α1D-adrenergic receptors is controlled by heterodimerization with α1B-adrenergic receptors. J. Biol. Chem..

[38] Hosoda C., Koshimizu T.-A., Tanoue A., Nasa Y., Oikawa R., Tomabechi T., Fukuda S., Shinoura H., Oshikawa S., Takeo S. (2005). Two α1-adrenergic receptor subtypes regulating the vasopressor response have differential roles in blood pressure regulation. Mol. Pharmacol..

[39] Vecchione C., Fratta L., Rizzoni D., Notte A., Poulet R., Porteri E., Frati G., Guelfi D., Trimarco V., Mulvany M.J. (2002). Cardiovascular influences of alpha1b-adrenergic receptor defect in mice. Circulation.

[40] Knappskog K., Botnar K., Kleinhapl J., Song J., El Ayadi A., Onarheim H., Guttormsen A.B., Golovko G., Wolf S., Almeland S.K. (2026). Vasopressor use in acute burn resuscitation: A retrospective study. Acta Anaesthesiol. Scand..

[41] Auchet T., Regnier M.-A., Girerd N., Levy B. (2017). Outcome of patients with septic shock and high-dose vasopressor therapy. Ann. Intensive Care.

[42] Jenkins C.R., Gomersall C.D., Leung P., Joynt G.M. (2009). Outcome of patients receiving high dose vasopressor therapy: A retrospective cohort study. Anaesth. Intensive Care.

[43] Martin C., Medam S., Antonini F., Alingrin J., Haddam M., Hammad E., Meyssignac B., Vigne C., Zieleskiewicz L., Leone M. (2015). Norepinephrine: Not too much, too long. Shock.

[44] Priyanka P., Chang C.-C.H., Chawla L.S., Kellum J.A., Clermont G., Murugan R. (2022). Vasopressor-resistant hypotension, combination vasopressor therapy, and shock phenotypes in critically ill adults with vasodilatory shock. Shock.

[45] Russell J.A., Walley K.R., Singer J., Gordon A.C., Hébert P.C., Cooper D.J., Holmes C.L., Mehta S., Granton J.T., Storms M.M. (2008). Vasopressin versus norepinephrine infusion in patients with septic shock. N. Engl. J. Med..

[46] Barrett L.K., Orie N.N., Taylor V., Stidwill R.P., Clapp L.H., Singer M. (2007). Differential effects of vasopressin and norepinephrine on vascular reactivity in a long-term rodent model of sepsis. Crit. Care Med..

